# 24 Months clinical prospective of proximal restorations with repeated preheating bulk fill composite up to ten cycles: randomized controlled trial

**DOI:** 10.1038/s41598-024-73200-6

**Published:** 2024-10-14

**Authors:** Islam Ibrahim, Heba Helal, Shereen Hafez Ibrahim, Mona Riad

**Affiliations:** 1 Conservative Dentistry Department, Faculty of Dentistry, Delta University for Science and Technology, Mansoura, Egypt; 2 Conservative Dentistry Department, Faculty of Dentistry, Cairo University, Cairo, Egypt

**Keywords:** Bulk fill resin composite, Composite restoration, Modified USPHS, Repeated preheating, Composite resin, Bonded restorations

## Abstract

This study aimed to clinically evaluate the performance of non-preheated bulk fill resin composite in class II cavities versus one, five and ten-time preheating cycles at 68 °C. Eighty-four recruited participants were randomly allocated into four groups (21 patients per group). One posterior Bulk-fill proximal restoration was performed for each participant according to the preheating cycles where in group I; teeth were restored with non-heated resin composite, group II: One preheating cycle of composite syringe was performed prior restoration, and group III: five preheating cycles while for group IV: Ten preheating cycles were performed. These restorations were clinically evaluated at 6, 12, 18 and 24 months. Statistical analysis was performed using one way ANOVA, with set p-value < 0.05. The results revealed that there was perfect agreement between both observers and observations WK (95% CI) [0.908 (0.780:1.000)] and [0.940 (0.824:1.000)], respectively. All restorations showed acceptable clinical performance with alpha and bravo scores. No replacement was required for any restoration. No statistically significant differences were detected between the control and intervention groups across all parameters during the various evaluation stages. It could be concluded that the clinical performance of ten cycles of repeated preheated bulk fill resin composite was equivalent to that of non-preheating resin composites after 24 months follow-up period, with great improvement in manipulation. Preheating of resin composite could be performed up to ten cycles prior to placement without any clinical adverse consequences on the performance of the restoration.

## Introduction

Resin composites are multipurpose materials that have the aesthetic qualities for anterior teeth and the mechanical qualities to be used for posterior tooth restorations. Adaptation to cavity walls and manipulation of the material are of primary concerns that are directly associated with material’s viscosity. Viscosity is primarily determined by the chemical composition of the resin composite, which includes the type of organic matrix and the kind, size, and concentration of filler particles^1^. The viscosity and mechanical properties of the resin composite increase with the monomers’ filler loading content and molecular weight, holding all other variables constant. However, highly viscous composite materials may not conform well to the cavity preparation procedure, potentially leading to poor marginal integrity and gap formation.

Resin composite placement by incremental technique has been considered as a standard approach in the field, but a new resin composite category, known as “bulk fill” has recently been developed^2^. These bulk fill materials are designed to have better light transmission qualities, a decreased polymerization shrinkage stress, and the ability to cure to a thickness of up to 4–5 mm. To minimize light scattering at the filler-matrix interface, these developments are due to either decreasing the filler quantity or increasing the filler size^3^.

The resin composite application process is particularly difficult for proximal class II restorations. Bulk fill resin composites are supplied by the producers to facilitate the application technique. The ability to apply more resin composite material in a single layer, up to a depth of 4 mm, adds to bulk-fill’s time-saving benefits. Unfortunately, due to its high viscosity, most bulk fill handling qualities are not ideal, particularly when restoring complex cavities^4^.

Highly viscous bulk-fill composites can contain air bubbles during packing which lead to the formulation of internal voids. Also, some studies revealed that high polymerization stresses long cavity walls especially in deep cavities restored with highly viscous bulk-fill resin composite compared to the multilayer technique^5^.

Preheating resin composite has been associated with several clinical advantages, particularly for high-viscosity resin composites, improves the material’s flowability and lowering its viscosity, allowing for better adaptability to cavity walls. Because preheating increases the mobility of radicals and monomers, which raises the degree of monomer conversion and improves the polymerization degree. When the monomers combine to form a polymer, the intermolecular distance changes from a van der Waals spacing of 0.3 to 0.4 nm to a covalent spacing of 0.15 nm, which causes the polymerization shrinkage of the resin composite. Most resin composites are methacrylate-based, which have a 2–5% polymerization shrinkage and may produce polymerization shrinkage stresses of up to 21 MPa^6,7^.

It appears that high viscosity bulk fill resin composites can be preheated to temporarily lower their viscosity to a level comparable to flowable composites while maintaining the benefits of heavily filled resin composites’ mechanical qualities^8^.

A highly cross-linked network containing covalent bonds between the polymer chains is formed during the resin composite polymerization phase. The crosslinking density and conversion rate increased quickly during photopolymerization, creating an endless network that leads the system viscosity to rise quickly. The initial transformation, from viscous liquids to elastic gels, is called gelation. Mobility affects free radicals more on larger molecules. Although there is less bimolecular termination, new polymerization extension centers can still be produced via initiation. Viscosity rises during the process, preventing auto-acceleration and monomer diffusion. Significant polymerization activities are blocked by vitrification. Preheating raises the temperature, which delays glassing. leading to higher final monomer conversion rate^9–11^.

The resin composite syringe is frequently used in clinical settings to restore several cavities. If preheating is used, the syringe will go through multiple heating cycles, so it is important to investigate the effects of repeated preheating on resin composite materials. The impact of several preheating cycles on post-gel polymerization shrinkage strain in bulk fill was assessed in previous study for up to three preheating cycles with no discernible variations in polymerization shrinkage strain^12^. The study’s findings justify the usage of preheated resin composites with performance equivalent to non-preheated. A clinical trial carried over 105 participants and evaluated the clinical performance of bulk fill posterior proximal restoration for one year follow up period. It was determined that the preheated resin composite performed better than the non-preheated ones^13^. Accordingly it was suggested that repeated preheating resin composite can improve clinical results and application simplicity without having any negative effects^14^.

Both the insertion method and the composites’ adaptation need to be improved, particularly in complex restorations to prevent the creation of gaps and voids. The preheating of conventional composite resin may lead to improved physicochemical properties by enhancing monomer conversion and increasing cross-linking in polymer formation. This process can also reduce the resin’s viscosity, making it suitable for use as a luting agent. Furthermore, increased flowability can minimize the incorporation of air bubbles and facilitate the material’s adaptation to cavity preparation walls^13^.

The reason of why preheating technique is not widely applicable by the dentists is that there are insufficient clinical trials to validate its usage in actual operating resin composite restorations. In addition to the concern that the preheating process could negatively impact the resin composite’s qualities. Studies that are clinically based have always been designed to address various factors that couldn’t be discussed in laboratory studies. The dynamic interrelation of oral environmental factors with dental restorations may interfere with clinical daily practice that amends some modifications to the routine dentists’ procedures performed with various restorative materials^15^. These clinical circumstances are functional forces, aqueous environment, acidic challenges, thermal stresses, enzymatic changes, and oral microflora. Even so, to attain a foreseeable and consistent dental service, emerging and adopting a standardized systematic convention for clinical assessment of the restorations was necessary such as The United State Public Health Service (USPHS) criteria and its modification^16^.

Owing to the limited clinical trials investigating the impact of repeated preheating of high viscous bulk fill resin composited in posterior restorations^11,13,16,17^, it was found valuable to assess and compare the clinical performance of posterior restorations with non- preheated bulk fill resin composite versus that with several preheating cycles to examine the null hypothesis, which claims that the clinical performance of posterior proximal cavities restored with non-preheated bulk fill resin composite versus after preheating for one, five, or ten times will not differ after 24 months follow up period. So the research question was in restoring posterior teeth, is there difference in clinical performance when using no heated, one, five and ten time preheated bulk fill resin composite using the modified United States Public Health Service criteria USPHS?

## Methodology

The armamentarium used in this randomized clinical trial were light curing posterior bulk-fill universal shade composite that allows the clinician to cure up to 4 mm layer (X-tra fil ^®^, VOCO, Cuxhaven, Germany), Universal adhesive bonding agent (Futurabond M+ ^®^, VOCO, Cuxhaven, Germany), and 37% Phosphoric acid etchant gel (Eco-etch ^®^, IvoclarVivadent, Liechtenstein). The composite heating device used was Therma-Flo™ Composite Warming Device (VISTA APEX, USA) to heat the resin composite to 68 °C to increase its flowability. The tested materials used, description, composition, lot number and manufacturer are presented in Table 1.

**Table 1:** Materials descriptions, compositions, manufacturer and lot number.

Commercial name	Description	Composition	Manufacturer	Lot number
Eco-etch	Acid etchant	Phosphoric acid (37%), thickness agent and color pigments	Ivoclar Vivadent, Schaan, Liechtenstein	Z04NW0
Futurabond M+	Universal adhesive system. Can be used with etch and rinse technique or self-etch technique. Secure adhesion to various materials such as metals, zirconium dioxide or aluminum oxide, as well as silicate ceramics, without additional primer.	MMA, Bis-GMA, UDMA, acid adhesive monomer, ethanol, water, catalyst	VOCO GmbH, Cuxhaven, Germany	2,250,318
X-tra Fil	Bulk-fill posterior resin composite Universal shade. Reduced working time Excellent physical properties.	Matrix: dimethacrylate (Bis-GMA, TEGDMA, UDMA) filler: Inorganic filler (Barium aluminum silicate, fumed silica, pigments) Filler content by weight :86%	VOCO GmbH, Cuxhaven, Germany	2,141,151

### Study setting

The protocol version of this clinical trial as well as the ethical requirements was revised by the Evidence Based Dentistry Committee (EBD) of the Conservative Dentistry Department and approved by the Research Ethics Committee of Faculty of Dentistry, Cairo University (CREC), with Reference number 20/3/22 and in accordance with the Declaration of Helsinki and its later modifications. The trial was registered in (www.clinicaltrials.gov) database in 20/5/2022, with unique identification number NCT05383768.

### Study design

**Fig. 1:**
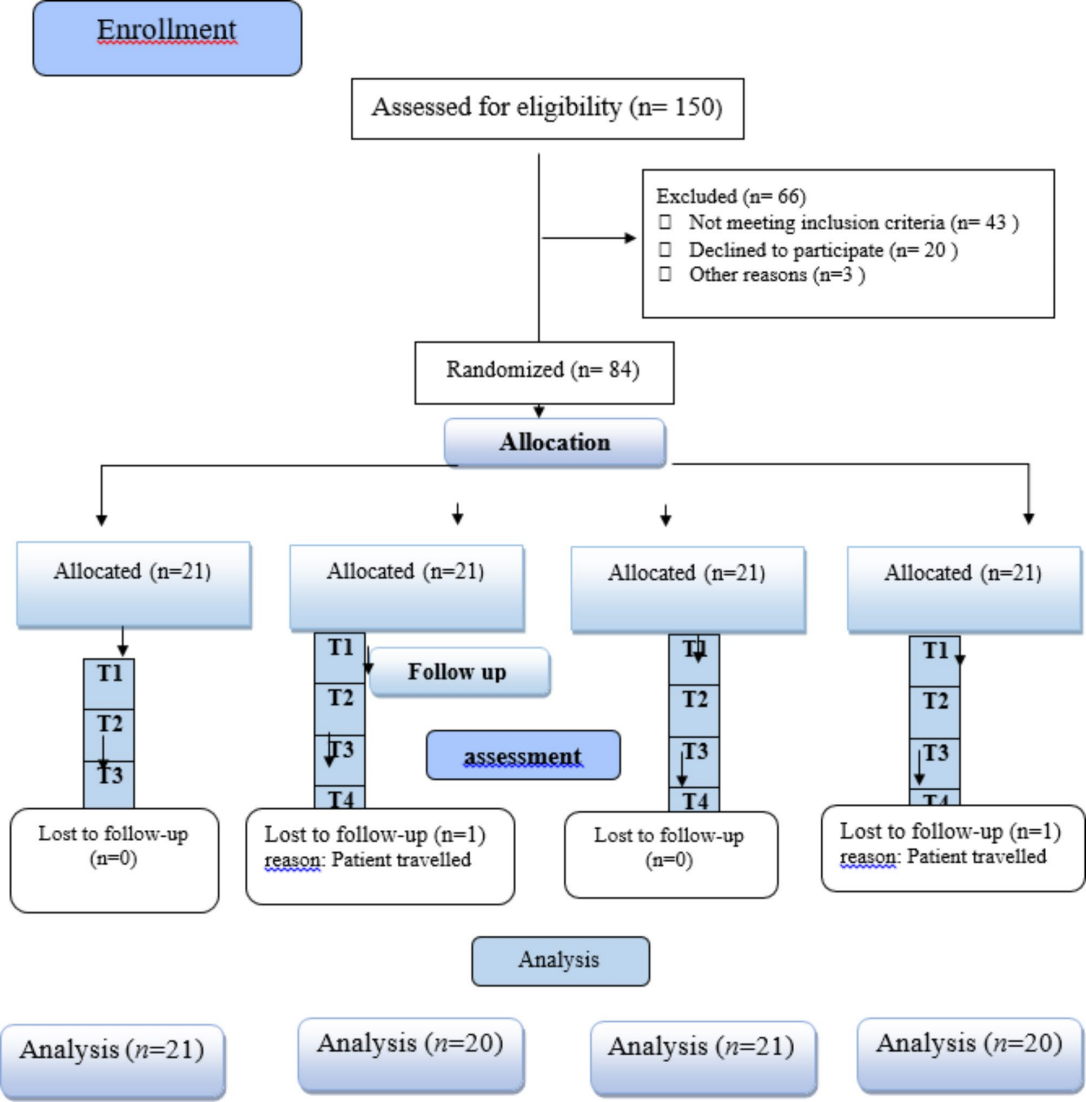
Flow chart of the study.

This study was double-blinded clinical trial where the outcome assessor and statistician were blinded The study was held in the clinic of the Conservative Dentistry Department. Recruitment of participants was performed between January 2022 and April 2022. The study design follows the CONSORT 2010 checklist, and the workflow was presented in CONSORT flow diagram (Fig. 1).

### Sample size calculation

A power analysis was designed to use marginal integrity scores as the primary outcome. According to the data of Tetric N Ceram Bulk fill published by (Alkurdi and Abboud 2016)^17^ the effect size w1 = (0.53) was figured. Whereas w2 = (0.7), w3 = (0.8) and w4 = (0.9), they were calculated upon expert opinion estimating Alpha scores = 85%, 90%, 95% and Bravo scores = 15%, 10% and 5%, respectively. By setting (5%) alpha (α) level of and (20%) Beta (β) level, the statistical power was 80%. Thus, it was suggested that the minimum estimated sample size was a total of 68 subjects that was increased to 84 subjects (i.e. 21 subjects per group) to compensate for the expected dropout rate of 25% during the follow up period Sample size calculation was performed using G*Power Version 3.1.9.2.

### Eligibility criteria and patient enrollment

One hundred fifty patients were screened, however, a total of 84 patients fulfilling eligibility criteria to be aged between (20–45) years old with a good oral health condition, having single primary ICDAS score 5 and D3 radiographic score class II cavities in vital carious molar with sound adjacent and opposing teeth were selected. Any cavity that exceeds 4 mm in depth would be excluded. Whereas, the excluded participants were with systemic disease, or who experienced allergic responses to materials used, or associated in another research, or orthodontic patients, lactating or pregnant females, or suffering from bruxism.

Each patient’s medical and dental history was carefully evaluated, and the history was recorded in their files. The diagnostic chart was filled out after dental and facial examination. The goal of the research, its procedures, safety precautions, benefits, and expected length of participation were all explained to each participant. Following that, before the trial started, patients had to complete an informed consent covering all ethical issues related to the experiment. Oral hygiene standards were promoted, and all participants were motivated. Polishing and scaling were performed as a prophylactic measures. Every dental issue was taken care of prior to the study starting.

### Randomization and blinding

**Fig. 2:**
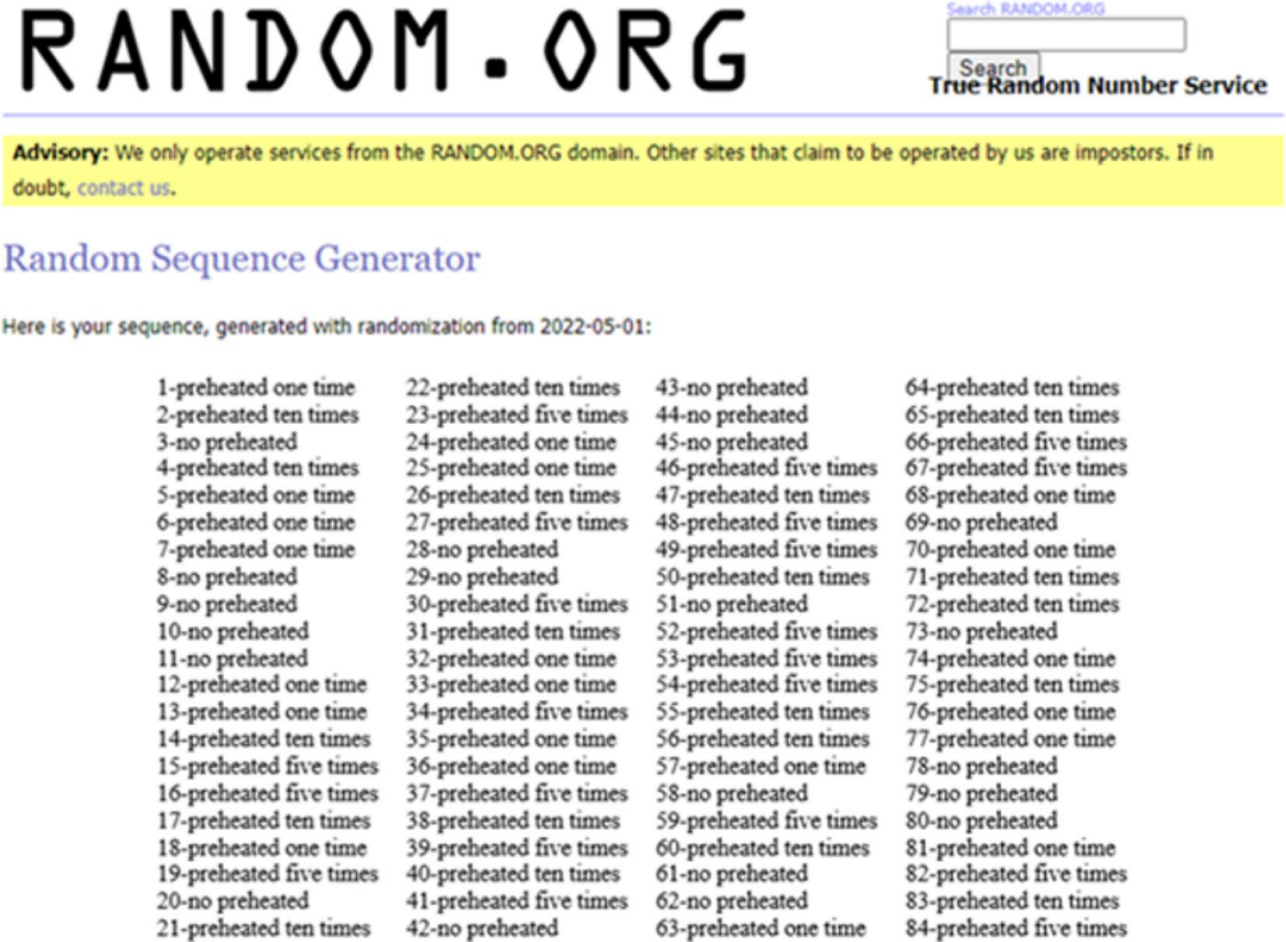
Simple randomization using (www.random.org).

A simple randomization was carried out using Random Sequence Generator (https://www.random.org). by generating numbers starting from 1 to 84, that were randomly divided into 4 groups representing the assignment of teeth either as an intervention or a control with a 1:1:1:1 allocation ratio where the group I: non-heated bulk fill resin composite was packed, group II: bulk fill resin composite syringe was preheated one time, group III: five preheating cycles were performed, and group IV: bulk fill resin composite was preheated for ten cycles. All patients and assessors were blinded while the operator was not blinded to performing the preheating cycles (Fig. 2).

#### Pre-operative assessment

Oral examination of the enrolled subjects was done using 4x prismatic dental loupes and light (Amtech, Wenzhou, China). Professional oral prophylaxis was performed using an ultrasonic scaler (Woodpeacker, Guangxi, China) and prophylactic paste (ALPHA-PROR prophylaxis paste, ALPHA-DENT, USA). The teeth were then thoroughly rinsed with water and then occlusal surfaces of the teeth were dried using air from the triple air-way syringe for 10 s. Preoperative clinical photographs for eligible participants were taken to assess the width of the lesion. Radiographs were taken to confirm the extent of the lesion and the health of the periodontal tissue. The pulp sensitivity was assessed with a cold test using dichlorodifluoromethane spray (endo ice) (Maquila, Brazil).

#### Cavity preparation and restorative procedures

A local anesthetic agent (ARTINIBSA 40 mg/ml + 0,01 mg/ml solution for injection(Inibsa, Barcelona, Spain) was administered to the patients preoperatively to control the patients’ discomfort during cavity preparation and restoration. Class II cavities were prepared according to the principles of minimally invasive dentistry. Cavity preparation was limited to just the removal of the carious lesion without any special retention aids (no undercuts) and no bevels. The cavities were cleared from any carious structure, and undermined enamel was removed using a fissured bur with round end (880 Oekodental, Germany) of 6 mm length and 1.4 mm width to the DEJ. Held in a high- speed contra-angle handpiece with water-cooling system (Alegra TE-95 LQ, W&H, Austria). All internal line angles were slightly rounded. Each bur was replaced after five cavities preparations.

The remaining soft caries was removed using a sharp excavator (Maillefer Dentsply Excavator Double-Ended, Switzerland) very conservatively leaving only smooth, hard dentinal bridge pulpally. Deep caries, if present was removed with a large rose head carbide bur (size 2) (MANI, INC, Japan) held in a low- speed contra-angle hand piece (WE 99 LED G, W&H, Austria) with a water-cooling system. Control of the depth of the prepared floor was done by visual inspection and confirmed by probing with a sharp probe to assess the consistency of the dentin. The walls of the cavities were finished using fine grit diamond stones (MANI, INC, Japan) with buccal and lingual walls were prepared parallel without occlusal convergence.

Teeth to be restored were isolated with medium-consistency rubber dam sheets (Dura Dam, TOP GLOVE, Malaysia) to ensure moisture control of the operative field and lack of contamination of the cavities with saliva, blood, or the sulcus fluid. Molar and /or premolar clamps were used for stabilization and isolation of the teeth (KSK Dentech, Tokyo, Japan). Class II cavities were restored using sectional matrix system (Palodent V3 Section matrix System, Dentsply Sirona, Germany). A suitable matrix and wedge were selected and stabilized by a separating ring to reestablish the interproximal contacts of the teeth.

All restorative procedures were applied according to the manufacturer’s instructions. A selective etching technique was used where the 37% phosphoric acid gel (Eco-etch ^®^, Ivoclar Vivadent, Liechtenstein) was applied to the enamel only for 15 s. The cavity was then thoroughly rinsed with air-water spray for 15 s and the excessive water was then removed using gentle air to avoid dehydration of dentin. Futurabond M+ (VOCO) was applied to cavities with micro brush (TPC Advanced Technology, Inc.) in a rubbing motion for 20 s followed by gentle air blow using oil-free air for 10 s then light cured for 20 s using 3 M Elipar curing unite(3 M, Neuss, Germany) with intensity output of 1200 mW/cm^2^, wavelength 430–480 and zero distance from the tooth surface.

##### For restoration with preheated X-tra bulk fill

Therma-flo TM Composite Warming Device (VISTA APEXInter-Med, Racine, USA) was used to warm X-tra fil bulk fill syringe (VOCO, GERMANY) that was labeled with the number of heating cycles. The warming device was turned on for 30 min, according to the manufacturer’s instructions, until it achieved a temperature of 68 °C. To get the syringe of resin composite to the same temperature as the warming device, it was placed into a heating chamber and left for five minutes.

**Fig. 3:**
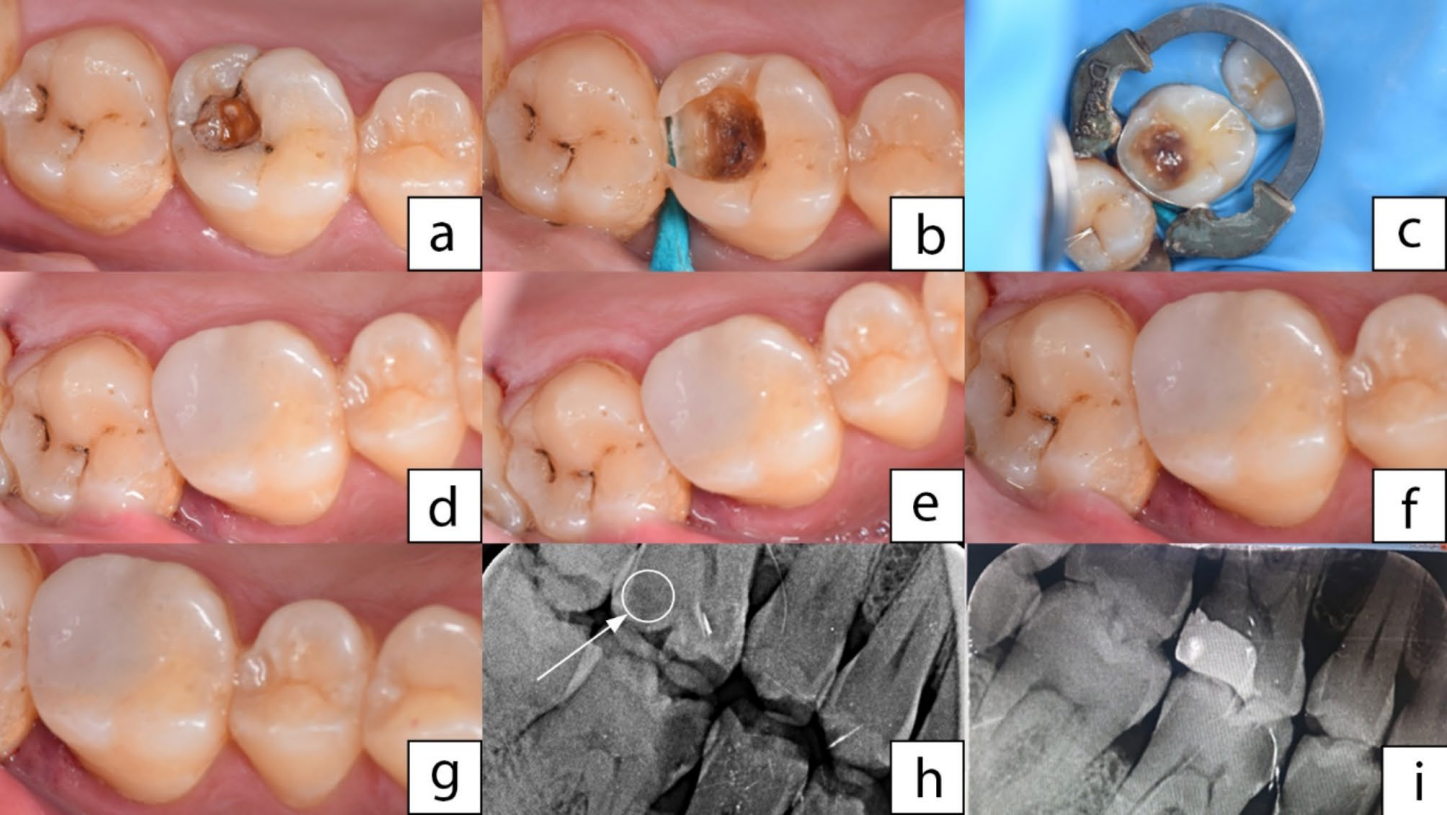
Full clinical casein tooth #16 restored by preheated bulk fill composite after ten heating cycles where (**a**) Preoperative clinical photograph for primary caries lesion in tooth #16, (**b**) Class II cavity preparation, (**c**) Matrixing procedure with Palodent V3 Section matrix System, (**d**) R estoration with bulk fill composite at 6months follow-up, e: follow-up after 12 months, (**f**) follow-up after 18 months, (**g**) follow up after 24 months with similar clinical performance to that at six months, (**h**) Bitewing preoperative radiograph with arrow pointing at the radiolucency in the distal surface of the tooth #16, for proximal caries lesion (**i**) Bitewing postoperative radiograph for composite restoration in the tooth #16 with proper internal adaptation to cavity walls and proper proximal contour.

The bulk fill syringe was removed from the apparatus, then preheated resin composite temperature was confirmed by k-type thermometer. Starting with the proximal box, a small layer of bulk fill was inserted to confirm good adaptation and no voids were between the resin composite and matrix band or tooth structure, followed by the remainder of the bulk fill layer. A composite plastic instrument was used to shape the occlusal surface and remove excess of resin composite. The restoration is light cured from the occlusal, buccal, and lingual sides for 20 s each. A total of 21 teeth were allocated for group II with X-tra fil (VOCO, Cuxhaven, GERMANY) which was preheated for one time. Another set of 21 teeth was allocated for group III with X-tra fil which preheated for five times. Furthermore, a set of 21 teeth was allocated for group IV with X-tra fil which preheated ten times (Fig. 3a-i).

##### For restoration with non-preheated X-tra fil bulk fill

A total of 21 teeth were allocated for group I with X-tra fill (VOCO, Cuxhaven, GERMANY) without preheating was applied In a same manner as mentioned before.

#### Occlusal and proximal adjustment

The occlusion was checked using 40 nm carbon articulating paper AccuFilmR II (ParkellR, Edgewood, NY, USA), to establish appropriate occlusal morphology and contacts. High spots were removed with yellow-coded stones (MANI, INC, Japan). The quality of the interproximal contacts and cervical adaptation were checked using dental floss (Essential Floss, Oral-B, Procter& Gamble, USA). Finishing of all restorations was performed with superfine tapered diamond stone yellow and white coded (MANI, INC, Japan) under water coolant. Restorations were polished with diamond diamond-impregnated two-stage spiral polishing system (EVE Diacomp Plus Twist, EVE Ernst Vetter GmbH; Pforzheim, Germany). Pink and grey polishing wheels in a mounted low-speed hand piece at a speed of 10,000 rpm were successively applied for 15 s under light pressure.

### Clinical evaluation of the restoration (outcome assessment)

Modified US Public Health Service (USPHS) criteria for dental restoration was the selected method to assess the clinical performance of the restoration^18^. Outcomes were evaluated and documented after 6, 12, 18 and 24 months in evaluation charts.

To ensure inter-examiner reliability, the study utilized a rigorous training program for the examiners at the beginning of the study. This involved repeated assessments of 20 posterior restorations using modified USPHS criteria. Live demonstrations were provided on a patient and instructed the assessors on how to examine and assess the outcomes. Each assessor was calibrated according to the modified USPHS criteria. Any conflicts in assessment between the examiners were resolved by the main supervisor, who made the final decision to obtain a single score for the evaluation of each restoration. This approach aimed to establish consistency and reliability in the assessment process.

Inter- and intra-observer reliability were analyzed using weighted kappa coefficient. There was an almost perfect agreement between both observers that was statistically significant WK (95% CI) 0.940 (0.824:1.000), *p* < 0.001and inter observer was 0.908 (0.780:1.000).

All evaluations were conducted under a dental operating light, using mirrors and dental explorers following the criteria listed in Tables 2 and 3. Restorations scored Alpha (A) and Bravo (B) are considered “Clinically acceptable”. The difference between Alpha and Bravo scores are only in degree and not in essence. However, restorations rated with Charlie (C) and Delta(D) scores had experienced an essential replacement.

**Table 2:** Modified USPHS criteria.

Criteria	Score	Characteristic
Post-operative sensitivity	Alpha	Absent
	Bravo	Present
Marginal adaptation	Alpha	No catch or discoloration between tooth structure and restoration
	Bravo	Detectable marginal discrepancy and catching clinically acceptable
	Charlie	The explorer penetrates crevice defect extended to the DEJ
	Delta	Marginal crevice, clinically unacceptable
Marginal discoloration	Alpha	No discoloration between tooth structure and restoration
	Bravo	Non penetrating marginal discoloration which can be polished away
	Charlie	Discoloration has penetrated margin in pulpal direction
	Delta	Strong discoloration in major parts of the margins, not removable
Secondary caries	Alpha	Restoration is in continuation of existing anatomic form
	Bravo	Visual evidence of dark discoloration not associated to the margins
	Charlie	Visual evidence of dark discoloration associated to the margins
Surface texture	Alpha	No surface defect
	Bravo	Minimal surface defect
	Charlie	Severe surface defect
Color matching	Alpha	The restoration matches the shade and translucency of the adjacent tooth
	Bravo	There is a mismatch in the shade and translucency, but within the normal range
	Charlie	The mismatch beyond the normal range of the tooth shades and translucency
Anatomical form	Alpha	Correct Contour
	Bravo	Slightly under-contoured
	Charlie	over or under-contoured
	Delta	Restoration fractured or mobile

**Table 3:** Clinical assessment criteria for the restorations.

Alpha (A)	Restoration has no errors as No defects Single pit	Accepted
Bravo (B)	Restoration has minor defects as: Marginal discoloration Discoloration of the restoration surface Ditching Limited wear	Accepted
Charlie (C)	Restoration has major faults Missing proximal contact Significant wear	Not accepted and the restoration should be replaced within the next few weeks.
Delta (D)	Fracture of the restoration Secondary caries Tooth fracture Pulpitis/persistent postoperative pain Loss of restoration	Not accepted and restoration must be renewed at once.

### Statistical analysis

Ordinal and categorical data were presented as frequency and percentage values. Categorical data were analyzed using Fisher’s exact test for determining whether the proportions of data described by two or more categorical variables are random. Ordinal data were analyzed using Kruskal-Wallis’s and Friedman’s tests for intergroup and intragroup comparisons, respectively. The Kruskal-Wallis H test is a rank-based nonparametric test that can be used to determine if there are statistically significant differences between two or more groups of an independent variable on a continuous or ordinal dependent variable. Friedman’s tests to determine differences between groups when the dependent variable being measured is ordinal. Demographic data were presented as mean and standard deviation values. They were tested for normality using Shapiro-Wilk’s test. They were found to be normally distributed and were compared using one-way ANOVA. The significance level was set at *p* < 0.05 within all tests. The statistical analysis was performed with R statistical analysis software version 4.3.3 for Windows^19^.

## Results

Intergroup and summary statistics for demographic data are presented in Table 4. The study was conducted on 84 equally and randomly allocated cases to each 4 tested group (i.e., 21 cases each). There were 9 males and 12 females with the mean age (30.79 ± 7.09) years in the no-heating group. In the one-time preheated group, there were 6 males and 15 females with a mean age of (31.43 ± 9.55) years. In the five-times preheated group, there were 10 males and 11 females with a mean age of (32.21 ± 8.13) years. Finally, in the ten-times preheated group, there were 11 males and 10 females with a mean age of (33.37 ± 10.42) years. There was no significant difference between tested groups regarding gender distribution (*p* = 0.447), age (*p* = 0.805), and treated teeth in each arch (*p* = 0.974). A total of two restorations were dropped out, one restoration was lost to followup at 6 months examination and another one restoration at 18 months examination with overall 97.6% retention rate.

**Table 4:** Intergroup and summary statistics for demographic data.

Parameter		Group				*p*-value
		No preheating	One-time preheated	Five-time preheated	Ten-time preheated	
Sex [n (%)]	Male	9 (42.86%)	6 (28.57%)	10 (47.62%)	11(52.38%)	0.447ns
	Female	12 (58.14%)	15 (71.43%)	11(52.38%)	10 (47.62%)	
Age (Mean ± SD) (years)		30.79 ± 7.09	31.43 ± 9.55	32.21 ± 8.13	33.37 ± 10.42	0.805ns
Treated arch [n (%)]	Mandibular molars	9 (42.86%)	11(52.38%)	11(52.38%)	11(52.38%)	0.974ns
	Maxillary molars	12 (58.14%)	10 (47.62%)	10 (47.62%)	10 (47.62%)	

ns; non-significant (*p* > 0.05).

### Postoperative sensitivity

After 6 months, a single case in each group had a bravo score, while at later intervals, all cases in all groups had an alpha score. Within all intervals, there was no significant difference between tested groups (*p* = 0.392), and within different groups, there was no significant difference between scores of different intervals (*p* = 0.392).

### Marginal discoloration

**Fig. 4:**
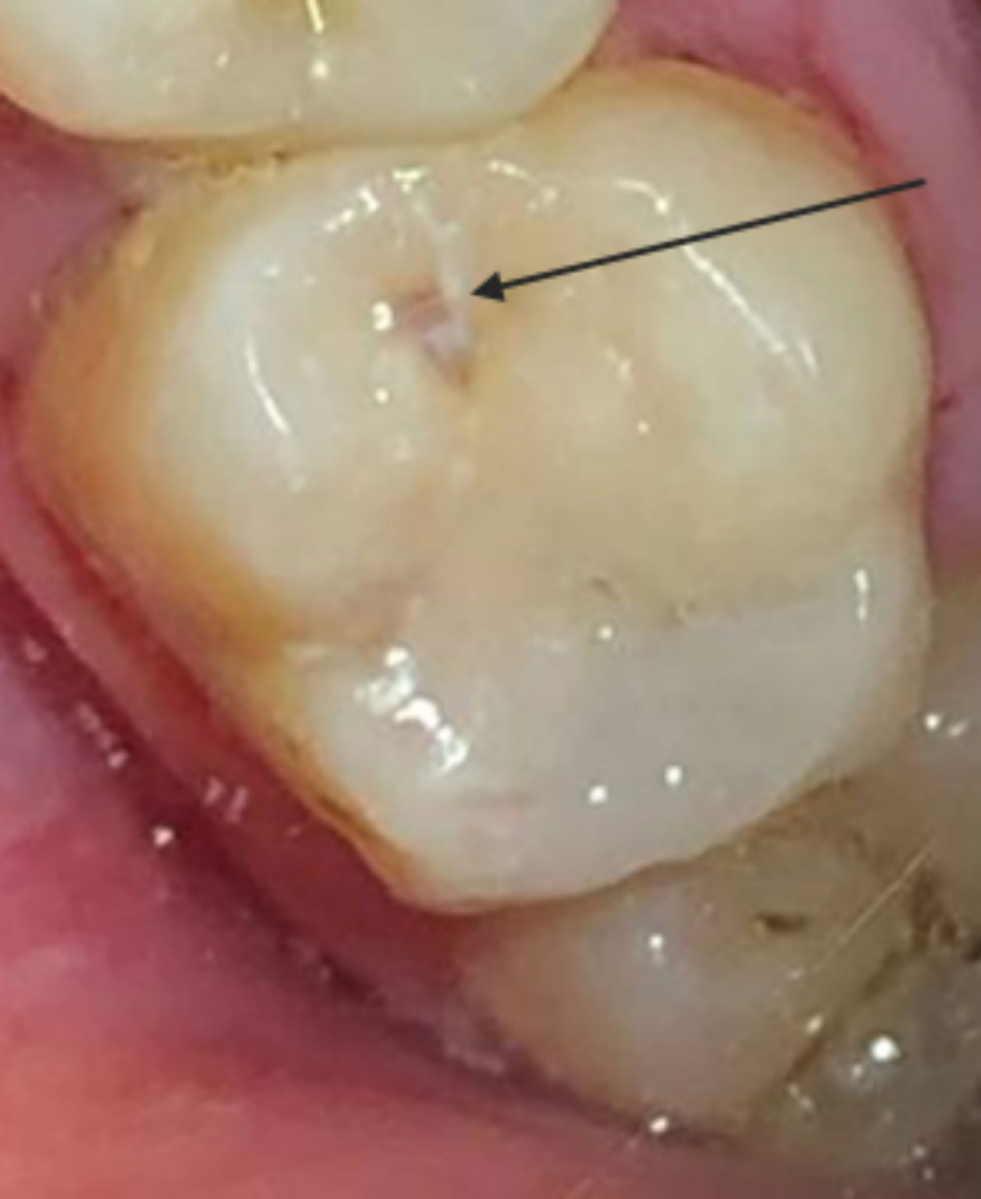
Marginal discoloration score Bravo in tooth #17 After 12 months follow-up period.

After 6 months, all cases in all groups had an alpha score. After 12 months, a single case within the no-preheating and ten-time preheated groups had a bravo score. Additionally, after 18 months, a single case scored bravo in the five-time preheated group. After 24 months, a single case in no preheating group and the five-time preheated groups had a bravo score. Within all intervals, there was no significant difference between tested groups (*p* > 0.05), and within different groups, there was no significant difference between scores of different intervals (*p* = 0.392) (Fig. 4).

### Surface texture

**Fig. 5:**
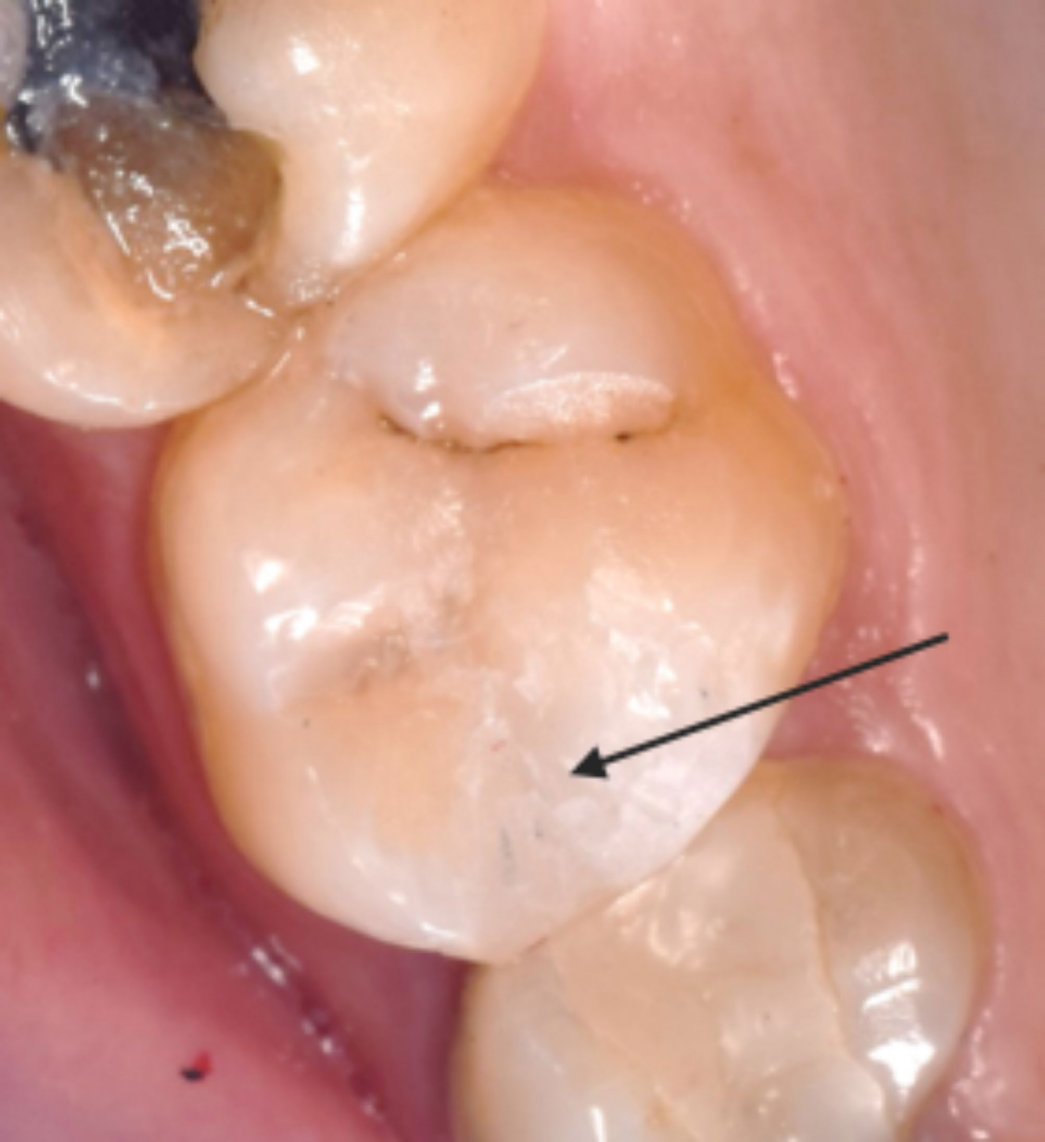
Surface texture score Bravo in tooth #26 after 6 months follow-up period.

After 6 months, all cases in the one-time preheated group had an alpha score, while other groups each had a single case with a bravo score. In all other intervals, all cases in all groups had an alpha score. Within all intervals, there was no significant difference between tested groups (*p* > 0.05), and within different groups, there was no significant difference between scores of different intervals (*p* = 0.392) (Fig. 5).

### Marginal adaptation, secondary caries, color matching, gross fracture, and anatomical form

Within all intervals, all cases of all groups had an alpha score (p not applicable). Frequency, and percentage values for all tested Modified USPHS criteria scores are presented in Table 5.

**Table 5:** The modified USPHS results after 24 months follow-up.

Category	Groups	6 months	12 months	18 months	24months	*p*-value
1. Post-operative sensitivity	No preheated	20 (95.24%)	21 (100.00%)	21 (100.00%)	21 (100.00%)	0.392ns
	One time preheated	20 (95.24%)	20 (100.00%)	21 (100.00%)	21 (100.00%)	0.392ns
	Five times preheated	20 (95.24%)	20 (100.00%)	21 (100.00%)	20 (100.00%)	0.392ns
	Ten times preheated	20 (95.24%)	20 (100.00%)	21 (100.00%)	20 (100.00%)	0.392ns
2. Marginal adaptation	No preheated	21 (100.00%)	21 (100.00%)	21 (100.00%)	21 (100.00%)	NA
	One time preheated	21 (100.00%)	20 (100.00%)	20 (100.00%)	20 (100.00%)	NA
	Five times preheated	21 (100.00%)	21 (100.00%)	21 (100.00%)	21 (100.00%)	NA
	Ten times preheated	21 (100.00%)	21 (100.00%)	20 (100.00%)	20 (100.00%)	NA
3. Marginal discoloration	No preheated	21 (100.00%)	20 (95.24%)	20 (95.24%)	20 (95.24%)	0.392ns
	One time preheated	21 (100.00%)	20 (100.00%)	20 (100.00%)	20 (100.00%)	NA
	Five times preheated	21 (100.00%)	21 (100.00%)	20 (95.24%)	20 (95.24%)	0.392ns
	Ten times preheated	21 (100.00%)	20 (95.24%)	20 (95.24%)	20 (95.24%)	0.392ns
4. Secondary caries	No preheated	21 (100.00%)	21 (100.00%)	21 (100.00%)	21 (100.00%)	NA
	One time preheated	21 (100.00%)	20 (100.00%)	20 (100.00%)	20 (100.00%)	NA
	Five times preheated	21 (100.00%)	21 (100.00%)	21 (100.00%)	21 (100.00%)	NA
	Ten times preheated	21 (100.00%)	21 (100.00%)	20 (100.00%)	20 (100.00%)	NA
5. Surface texture	No preheated	21 (100.00%)	20 (95.24%)	20 (95.24%)	20 (95.24%)	0.392ns
	One time preheated	21 (100.00%)	20 (100.00%)	20 (100.00%)	20 (100.00%)	NA
	Five times preheated	21 (100.00%)	21 (100.00%)	21 (100.00%)	2(100.00%)	0.392ns
	Ten times preheated	21 (100.00%)	20 (100.00%)	20 (100.00%)	20 (100.00%)	0.392ns
6. Color matching	No preheated	21 (100.00%)	21 (100.00%)	21 (100.00%)	21(100.00%)	NA
	One time preheated	21 (100.00%)	20(100.00%)	20 (100.00%)	20 (100.00%)	NA
	Five times preheated	21 (100.00%)	21 (100.00%)	21 (100.00%)	21 (100.00%)	NA
	Ten times preheated	21 (100.00%)	21 (100.00%)	20 (100.00%)	20(100.00%)	NA
7. Gross fracture	No preheated	21 (100.00%)	21 (100.00%)	21 (100.00%)	21 (100.00%)	NA
	One time preheated	21 (100.00%)	20 (100.00%)	20 (100.00%)	20 (100.00%)	NA
	Five times preheated	21 (100.00%)	21 (100.00%)	21 (100.00%)	21 (100.00%)	NA
	Ten times preheated	21 (100.00%)	21 (100.00%)	20(100.00%)	20(100.00%)	NA
8. Anatomical form	No preheated	21 (100.00%)	21 (100.00%)	21 (100.00%)	21 (100.00%)	NA
	One time preheated	21 (100.00%)	20 (100.00%)	20(100.00%)	20(100.00%)	NA
	Five times preheated	21 (100.00%)	21 (100.00%)	21 (100.00%)	21(100.00%)	NA
	Ten times preheated	21 (100.00%)	21 (100.00%)	20 (100.00%)	20 (100.00%)	NA

NA: Not Applicable, ns; non-significant (*p* > 0.05).

## Discussion

Throughout the different evaluation stages, no significant changes were found between the control and intervention groups in terms of any criteria. Thus, the null hypothesis was accepted.

The resin composite syringe is frequently used in clinical settings to restore several cavities. If preheating is used, the syringe will go through multiple heating cycles, so it is important to investigate the effects of repeated preheating on resin composite materials. The impact of several preheating cycles on post-gel polymerization shrinkage strain in bulk fill was assessed in previous study for up to three preheating cycles with no discernible variations in polymerization shrinkage strain^10^. The study’s findings justify the usage of preheated resin composites with performance equivalent to non-preheated. A clinical trial carried over 105 participants and evaluated the clinical performance of bulk fill posterior proximal restoration for one year follow up period. It was determined that the preheated resin composite performed better than the non-preheated ones^16,17^. Accordingly it was suggested that repeated preheating resin composite can improve clinical results and application simplicity without having any negative effects.

Because randomized clinical trials offer a standardized methodology for guaranteeing reliability and improved clinical validity, they are an essential tool for the clinical assessment of new materials and procedures.

In this clinical trial, the regular bulk fill resin composite material used as the control group was Xtra fil (Voco, Cuxhaven, Germany). This bulk fill packable posterior resin composite has a shrinkage of 1.7% due to a blend of new multi-hybrid filler technology with an innovative initiator system, resulting in a filler material with minimal polymerization shrinkage and outstanding depth of cure. Additionally, pre-heating of resin composite is utilized to enhance material handling properties. Raising the temperature of resin composites before curing reduces their viscosity, leading to improved marginal adaptation and better wetting of the composite resin to the cavity walls^20^.

It is important for an ideal restorative material to possess specific characteristics such as dimensional stability, wear resistance, adequate strength for chewing forces, biocompatibility, and antibacterial properties. Additionally, it should be easy to use for quick placement and should also have aesthetic qualities like color stability and stain resistance. The current study focused on evaluating the functional outcomes of restorations primarily based on anatomical form, with biological and aesthetic aspects considered as secondary outcomes.

The study started with 84 participants, 82 participants who completed the 24-month follow-up with a 98% retention rate. It is very important to use simple, objective, and reliable criteria in clinical trials to assess the clinical performance of restorative materials. The USPHS criteria are the most used for this purpose. This approach can provide valuable insights into the initial effectiveness of the tested techniques^16^.

No significant differences in the clinical performance of the restorations were found based on gender, age, or teeth distribution among the participants. Regarding post-operative sensitivity results showed that after 6 months, a single case in each group had a bravo score, while at later intervals, all cases in all groups had an alpha score could be attributed to postoperative sensitivity was correlated to preoperative sensitivity^21^. It is understandable that dentists may have reservations about using preheated resin composites due to concerns about potential damage to pulp tissue and increased postoperative sensitivity. However, previous clinical studies have not supported these concerns. In these studies, researchers investigated the impact of preheating resin composite on postoperative sensitivity, particularly in posterior restorations.

The results showed no significant difference in postoperative sensitivity between restorations where the resin composite was preheated and those where it was not. This with the agreement with study by Elkady et al., 2024 which used VAS scores to assess the effect of repeated resin composite preheating in patient postoperative sensitivity and came to conclusion that preheating the bulk-fill resin composite many times before curing did not cause postoperative hypersensitivity^21^. This suggests that preheating composite resin may not contribute to greater postoperative sensitivity as previously thought^1,22^.

The absence of secondary caries in the evaluated restorations at various time points can be attributed to the excellent marginal adaptation of the restorations. Marginal adaptation is a critical factor in the longevity of dental restorations and is primarily influenced by the polymerization shrinkage of the resin composite and the type of adhesive used. Both of these factors play a significant role in determining the clinical success of restorations^23^. This result with agreement with previous clinical research done by (Elkady et al. 2024)^24^.

Marginal discolorations observed may be due to the polymerization shrinkage of the resin composite which leads to formation of leaky gaps which are due to some individual factors as the eating habits, smoking and drinking tea and coffee, as well as some factors related to the restoration as flashes or excess of restorative material, they all can contribute to increase the marginal discoloration^25^. In study compared room temperature and preheated resin composite restoration marginal staining and surface staining were significant for both groups after 2 years compared to base line scores^26^.

The marginal integrity of the preheated groups was satisfactory and on comparable to that of the non-preheated group. In comparison the clinical trial by (El Kady et al. 2024) which found that nonprehated group showed inferior results compared to preheated groups^24^. The benefits of preheating resin composite are confirmed by the outcomes of in-vitro studies. This allows the clinician to take advantage of decreasing the viscosity of resin composite without modifying the mechanical characteristics or restoration longevity. Preheating resin composite enhanced its physical characteristics, decreased polymerization shrinkage (1.7–3.1%), accelerated cure, and raised the degree of monomer conversion, all of which enhanced the resin composite’s marginal adaptation^20^.

Regarding the surface texture results, at every follow-up interval, there was no statistically significant difference between the different groups that were compared. This could be because resin composite technology has advanced significantly. Bisphenol A-glycidyl methacrylate is the most often used resin monomer in bulk fill resin composite structures. Compared to other types of monomers, it is less flexible and more rigid. Furthermore, both groups received the same finishing and polishing protocols, which ensured a durable surface finish and polish^27^. In comparison to the study conducted by Elkaffass et al. 2022 who found that the surface roughness of preheated Z350 XT resin composite was higher than non preheated resin composite^28^.

Regarding the color matching results show, at every follow-up interval, there was no statistically significant difference between the different groups that were compared this results in agreement with previous clinical research by (Favoreto et al. 2022)^1^ that found no statistically significant difference between non heated and preheated restorations in non-carious cervical lesions (NCCLs) that is may be due to proper finishing an polishing protocol used.

Regarding retention and gross fracture, the results showed that within all intervals, all cases in all groups had an alpha score. Like other previous clinical trials where the heated groups had no negative effects on these aspects, fracture and retention did not exhibit any inferior results. This could be because the effectiveness of these requirements depends on a number of factors, including the adhesive method, cavity design, in addition to the mechanical characteristics of the resin composite^4^.

After monitoring the clinical performance of all the examined restorations for 24months, they were deemed satisfactory, receiving either “Alpha” or “Bravo” ratings for all evaluated criteria. Most of them were rated as “Alpha.” The primary outcome was marginal integrity, while marginal discoloration, secondary caries, and post-operative sensitivity were secondary outcomes. No notable significant differences were detected between the control and intervention groups across all parameters during the various evaluation stages. Therefore, the null hypothesis was supported and accepted, indicating no statistically significant differences in clinical performance between the control and the different groups of intervention. Limitation of the study:

Randomized clinical trials represent one of the most important ways to assess the value of materials and methods since they give a uniform course that assures dependability and enhances the validity of any clinical finding. Such studies minimize natural tooth variability-induced bias by randomly distributing teeth into groups.

The limitations of this study were represented by the clinical circumstances encountered with different oral environmental conditions, preheating temperature and type of resin composite used. Diversity of the oral condition regarding patient occlusal forces, different between upper and lower molars teeth, difference between first and second molar teeth mesial and distal proximal cavities. To minimize the cofounders and bias that may result from inherent tooth variability by randomly allocating teeth to various groups, follow restrict inclusion and exclusion criteria. Furthermore, having a single operator handle all restorative operations is advantageous as it enables a more controlled comparison.

## Conclusions

Under these limitations and based on the current findings, it could be settled that when employing bulk fill without preheating and after repeatedly preheating for one, five, and ten times in the restoration of posterior teeth, there was almost similar clinical performance of the restoration following a 24-month follow-up with great enhancement in resin composite manipulation.

### Clinical relevance

Bulk fill resin composite syringes could be preheated up to ten times prior to placement to enhance the clinical application, minimize the viscosity of the material and improve their adaptation to the cavity walls without any adverse effect on the clinical performance of the final restoration.

### Recommendations

1. Preheating of composite resin prior to placement could be recommended to obtain more adequate manipulation properties.
2. To validate the current findings and track the clinical performance over an extended duration of clinical service, longer follow-up clinical trials are recommended. Also, different bulk fill resin composite brands should be tested with different preheating temperatures.
3. More laboratory investigations are required to assess and monitor the structural changes that might occur because of repeated preheating cycles that could affect the chemical, mechanical, physical, rheological, and optical properties of the material.

## Data Availability

The data that support the findings of this study are available from the corresponding author, on reasonable request.

## Abbreviations

USPHS: Modified United States Public Health Service criteria
EBD: Evidence Based Dentistry Committee
CREC: Research Ethics Committee of Faculty of Dentistry, Cairo University
ANOVA: Analysis of variance
NCCLS: Non-carious cervical lesions
nm: Nanometer
mm: Millimeter
